# Rheumatoid arthritis is an independent risk factor for multi-vessel coronary artery disease: a case control study

**DOI:** 10.1186/ar1775

**Published:** 2005-06-29

**Authors:** Kenneth J Warrington, Peter D Kent, Robert L Frye, James F Lymp, Stephen L Kopecky, Jörg J Goronzy, Cornelia M Weyand

**Affiliations:** 1 Division of Rheumatology, Mayo Clinic, Rochester, MN, USA; 2 Division of Cardiovascular Diseases, Mayo Clinic, Rochester, MN, USA; 3 Mayo Alliance for Clinical Trials and the Mayo Clinic, Rochester, MN, USA; 4 Division of Biostatistics, Mayo Clinic, Rochester, MN, USA; 5 Emory University School of Medicine, Atlanta, GA, USA

## Abstract

The risk for cardiovascular (CV) disease is increased in rheumatoid arthritis (RA) but data on the burden of coronary atherosclerosis in patients with RA are lacking. We conducted a retrospective case-control study of Olmsted County (MN, USA) residents with RA and new-onset coronary artery disease (CAD) (*n *= 75) in comparison with age-and sex-matched controls with newly diagnosed CAD (*n *= 128). Angiographic scores of the first coronary angiogram and data on CV risk factors and CV events on follow-up were obtained by chart abstraction. Patients with RA were more likely to have multi-vessel coronary involvement at first coronary angiogram compared with controls (*P *= 0.002). Risk factors for CAD including diabetes, hypertension, hyperlipidemia, and smoking history were not significantly different in the two cohorts. RA remained a significant risk factor for multi-vessel disease after adjustment for age, sex and history of hyperlipidemia. The overall rate of CV events was similar in RA patients and controls; however, there was a trend for increased CV death in patients with RA. In a nested cohort of patients with RA and CAD (*n *= 27), we measured levels of pro-inflammatory CD4^+^CD28^null ^T cells by flow cytometry. These T cells have been previously implicated in the pathogenesis of CAD and RA. Indeed, CD4^+^CD28^null ^T cells were significantly higher in patients with CAD and co-existent RA than in controls with stable angina (*P *= 0.001) and reached levels found in patients with acute coronary syndromes. Patients with RA are at increased risk for multi-vessel CAD, although the risk of CV events was not increased in our study population. Expansion of CD4^+^CD28^null ^T cells in these patients may contribute to the progression of atherosclerosis.

## Introduction

Inflammation plays a central role in the pathogenesis of atherosclerosis [1,2]. Markers of inflammation, such as C-reactive protein, are predictive of future cardiovascular (CV) events in healthy individuals and may be useful in identifying patients with coronary artery disease (CAD) who are at risk for recurrent CV events [3,4]. Atherosclerotic plaque is a complex inflammatory lesion characterized by an infiltrate of macrophages and T cells [1]. Intraplaque immune cells are activated and involved in mediating tissue injury [5]. T-cell cytokines can drive macrophage activation in atherosclerotic lesions and can also regulate the acute-phase response [1]. Indeed, T cells in patients with acute coronary syndromes (ACS) are skewed toward the production of interferon (IFN)-γ, a potent monocyte activator largely derived from a distinct subset of CD4^+ ^T cells [6,7] that, in contrast to classic CD4^+ ^helper T cells, lacks the costimulatory molecule CD28 [8]. CD4^+^CD28^null ^T cells are clonally expanded in ACS and invade the unstable atherosclerotic plaque [9]. Moreover, CD4^+^CD28^null ^T cells have cytotoxic capability, can effectively kill endothelial cells *in vitro*, and may contribute to endothelial cell injury in coronary plaque [10].

Expansion of CD4^+^CD28^null ^T cells was initially described in patients with rheumatoid arthritis (RA), a chronic autoimmune disease of unknown etiology [11]. RA is characterized by chronic inflammation and hyperplasia of synovial tissue. More importantly, it is a quintessential systemic disease that can manifest in most major organ systems [12]. T cells play a central role in the immunopathogenesis of RA and are the key regulators of the chronic destructive joint lesions [13]. In addition, patients with RA have abnormalities in T-cell homeostasis that affect the entire pool of T cells [14,15]. One of the consequences of dysregulated T-cell homeostasis is the emergence of large clonal CD4^+^CD28^null ^T-cell populations that are autoreactive and cytotoxic, and infiltrate synovial tissue [15]. The highest frequency of CD4^+^CD28^null ^T cells is found in severe RA, particularly in patients with rheumatoid vasculitis [11,16]. When the inflammatory process in RA spreads to extra-articular sites, such as mid-size arteries and capillaries, morbidity and mortality are clearly increased [17].

Because the chronic inflammatory process and immune dysregulation in RA have features in common with those involved in atherosclerosis, they could predispose patients with RA to accelerated CAD. Several studies have documented an increased risk of atherosclerosis and myocardial infarction in patients with RA [18-20]. In addition, RA is associated with a reduced life expectancy, primarily because of excessive deaths from CV disease [21-25]. RA is a heterogeneous disease, and the disease phenotype itself is predictive of mortality; patients with more severe clinical disease have higher mortality rates [26]. Overall mortality is also increased in patients who are positive for the autoantibodies, rheumatoid factors [27,28]. In addition, the extent of inflammation in RA has been linked to an increased risk of CV mortality [29]. The number of swollen joints, independent of traditional CV risk factors, is predictive of CV-related deaths among Pima Indians with RA [30]. The strongest association with increased CV mortality is seen in patients with extra-articular manifestations of RA [17].

Our study demonstrates that patients with RA have significantly more multi-vessel coronary disease by angiography compared with patients with CAD but no RA. This finding may, at least in part, result from the expansion of proinflammatory CD4^+^CD28^null ^T cells that have previously been shown to play a role in the pathogenesis of CAD.

## Methods

### Data source

The Rochester Epidemiology Project maintains the medical records of patients from Mayo Clinic Rochester, MN, USA, which is the major referral center for secondary and tertiary care, including coronary angiography, in Olmsted and surrounding counties. The complete medical records of each study subject were retrieved and reviewed. The study was approved by the Mayo Foundation Institutional Review Board and patient consent was obtained.

### Patient population

We studied the medical records of patients from Olmsted and surrounding counties. Patients with RA who developed CAD between January 1985 and December 1998 and who had a coronary angiogram at Mayo Clinic Rochester were recruited for the study. Inclusion criteria were: diagnosis of RA according to the 1987 American College of Rheumatology criteria [31]; diagnosis of ischemic heart disease (ischemic heart disease criteria were anginal pain or anginal equivalent symptoms occurring with exercise, relief by rest or nitroglycerin. If occurring at rest, then symptoms relieved with nitroglycerin); and coronary angiography performed at Mayo Clinic for evaluation of CAD within the first 12 months of disease. Mayo Clinic is the only provider of invasive cardiac care in Olmsted County.

Exclusion criteria were: congestive heart failure without ischemic heart disease; CAD present for more than 12 months prior to the first angiogram at Mayo Clinic (to ensure that all angiograms reflected the status of the patient at onset of CAD symptoms); and prior coronary artery bypass graft (CABG), myocardial infarction (MI) or percutaneous transluminal coronary revascularization (PTCR). Death during the study period 1985 to 1998 was not an exclusion criterion.

Residents of Olmsted and surrounding counties who were seen at Mayo Clinic between January 1985 and December 1998 and diagnosed with CAD during the study period were used as control subjects. The original study design was to match three controls for each of the 75 RA cases for age, sex and visit date. Controls were matched for age at diagnosis of CAD and year of onset in order to control for shifts in practice and diagnostic patterns. Using these criteria, we were able to identify 130 controls, therefore some cases had one control and others had two controls. After matching, two patients were excluded for prior CABG, MI, PTCR or no angiogram at Mayo Clinic, resulting in 128 controls. Inclusion/exclusion criteria were identical except that a diagnosis of RA was an additional exclusion criterion. The controls were from the local, stable population of Olmsted and surrounding counties. The only rheumatology practice in this geographic area is at Mayo Clinic. The investigators reviewed the entire Mayo medical record (including the master sheet listing all diagnoses for the patient throughout his/her lifetime) for each control. We are confident that symptoms or clinical manifestations of RA would have been recorded in the patients' charts. The diagnosis for RA was based on a rheumatologist's evaluation.

### Coronary angiogram data

Angiographic data was retrieved from the Mayo Clinic coronary care unit database. Mayo Clinic cardiologists who performed the study and read each angiogram were not directly involved in each patient's medical care and, therefore, not typically informed of the patient's concurrent medical problems. Vessel involvement was defined as >50% stenosis for the left main coronary artery and >70% stenosis for the left anterior descending, right coronary and circumflex arteries. For each involved vessel, the patient received a score of 1.

### Ischemic risk factors

Ischemic heart disease risk factors were ascertained by medical record review and were defined as follows: cholesterol ever ≥ 200 mg/dl and/or treated by a physician for hypercholesterolemia, hypertension under treatment, smoking history (yes or no), and type I or type II diabetes. We have selected variables in our analysis where the data sets were largely complete and where there was not a difference between the two patient cohorts in terms of missing data. Variables for which we did not have a complete dataset (such as quantitative details of smoking history) were excluded.

### Immune markers

In a nested study, all RA+CAD subjects who were alive at the time of the study were contacted by mail and invited to participate in the immune marker analysis. Of these, 27 individuals consented to blood donation, and peripheral blood was obtained for T-cell phenotyping (*n *= 27). This patient subgroup had similar demographic characteristics as the whole RA+CAD cohort. Mean age for the subgroup was 69.2 ± 8.2 years, 58% were male and 87% were rheumatoid factor-positive. RA disease duration was also similar (15.9 ± 9.9 years). Blood samples were also obtained from controls who were classified as having had stable angina (*n *= 24). Results were compared with 22 patients with unstable angina type Braunwald IIIb, from a previous study [6].

Peripheral blood mononuclear cells were isolated by density gradient centrifugation with Ficoll-Paque (Amersham Biosciences, Arlington Heights, IL, USA). Cell surface staining was performed using anti-CD4^FITC ^and anti-CD28^PE ^antibodies (BD Biosciences, San Jose, CA, USA). Data was collected on a FACScan flow cytometer (Becton Dickinson, San Jose, CA, USA), and the frequencies of CD4^+ ^T-cell subsets were calculated using WinMDI software (Joseph Trotter, Scripps Research Institute, La Jolla, CA, USA).

### Statistical methods

All time references are based on the index angiogram. For each patient, the following variables were considered: RA diagnosis; number of diseased vessels (0, 1, 2 or 3); age and sex; history of diabetes, hypertension, hyperlipidemia or smoking; time to death, follow-up angiogram, follow-up CABG or follow-up MI; and time to first event [death, CV death, angiogram, CABG or MI]. In all models, the number of diseased vessels was treated as a four-level categorical variable.

The first component of the analyses was the exploration of baseline factors associated with RA diagnosis and number of diseased vessels. These data were cross-tabulated with each other and with each of the other baseline variables. Chi-square tests or Wilcoxon rank-sum tests were performed as appropriate. A multiple logistic regression model, with RA diagnosis as the dependent variable, was constructed using forward selection, with smallest *P *< 0.05 as the entry criterion. Additionally, a multiple ordinal logistic regression model, with number of diseased vessels as the dependent variable, was constructed using forward selection, with smallest *P *< 0.05 as the entry criterion.

The second component of the analyses was the exploration of follow-up events. RA diagnosis was cross-tabulated with the various follow-up events: time to death, CV death, time to follow-up angiogram, follow-up CABG, follow-up MI and first event (death, angiogram, CABG or MI). The cases and controls were defined during a specified time interval and each subject was followed for subsequent events. Therefore, the study group is a sample from a cohort of patients with CAD, some with RA and some without RA, allowing us to use Cox proportional hazards models for each of the five follow-up endpoints and for RA diagnosis. Adjusted Cox proportional hazards models were fit by adding the number of diseased vessels and hyperlipidemia as covariates. For time to death, a Cox model was fit for each of the other baseline variables.

Kaplan-Meier survival plots were constructed for time to death as a function of each baseline variable. Statistical analyses were performed using SAS Release 8.2 (TS2M0) for UNIX (SAS Institute Inc, Cary, NC, USA) and S-PLUS 2000 Professional Release 2 for Windows (Insightful Corp, Seattle, WA, USA). CD4^+^CD28^null ^T-cell percentages were compared using the Mann-Whitney rank sum test.

## Results

### Demographic characteristics and CAD risk factors

The study population consisted of 79 patients with RA who had coronary angiography performed at Mayo Clinic for CAD symptoms. Four were excluded because of history of CAD diagnosis and intervention prior to evaluation at Mayo. The control population consisted of 130 individuals with CAD but no history of RA, two of whom were excluded because of a history of prior CV events. The analyzed dataset consisted of 75 cases and 128 controls.

There was no significant difference in the percentage of individuals with traditional risk factors for CAD, including diabetes, hypertension, hyperlipidemia or smoking history in the study group when compared with controls (Table 1). Because age and sex were incorporated in the matching, there was no difference between the two groups.

### Characteristics of cases with RA and CAD

Table 1 includes characteristics of the patients with RA. Average age at onset of RA was 55 years and the average disease duration was 17.6 years. Approximately 90% of the cases were rheumatoid factor-positive and 53% had nodular disease. One-fifth of the group had extra-articular disease manifestations including vasculitis, rheumatoid lung disease, pericarditis, Felty's syndrome, neuropathy and scleritis. Use of corticosteroids and disease modifying therapy was fairly typical of patients with long-standing RA.

### Coronary artery involvement

There was a statistically significant difference in the distribution of the number of involved vessels in the two groups. More patients with RA had significant coronary artery involvement compared with controls (*P *= 0.002, chi-square = 14.6866, df = 3; Table 2). Only 4% of the patients with RA had no significant vessel involvement compared with 23% for the control patients.

### RA is an independent risk factor for increased CAD severity

Table 3 shows the ordinal logistic regression model results for number of diseased vessels. One model includes only RA diagnosis and the other also includes the covariates added via the forward selection procedure. These variables were added because they were related to the number of diseased vessels. RA remains a significant risk factor for multi-vessel disease after adjustment for age, sex, and history of hyperlipidemia. The odds ratio of RA diagnosis for an increase of one diseased vessel is 1.73 (95% CI: 1.03, 2.91) unadjusted and 1.97 (95% CI: 1.15, 3.36) adjusted.

### CV events

Because RA was associated with more severe coronary disease, we investigated whether this resulted in an increased incidence of CV events and/or premature mortality. Follow-up data for CV events including CV death, death from any cause, CABG, MI and PTCR was recorded by review of the medical record. Mean duration of follow-up was 62.4 months for the RA and CAD cases and 57.8 months for the CAD-only controls. The mean time from CAD onset to occurrence of a CV event (MI, CABG or PTCR) was 18 months and the mean time to death from any cause was 63 months. The risk of non-fatal CV events did not differ significantly between cases and controls. However, RA and CAD cases tended to have increased all-cause mortality (32%) compared with CAD only controls (18%); unadjusted risk ratio = 1.6 (95% CI: 0.9–2.9). In particular, the risk of CV death was increased in RA and CAD cases (17%) compared with CAD-only controls (7%); unadjusted hazard ratio (HR)=2.2 (95% CI: 0.95–5.2). However, neither comparison reached significance.

Table 4 shows raw counts and Cox model results for various follow-up endpoints. Of the 203 patients in the study, 47 died, 52 had follow-up CABG events, 71 had follow-up MI, 74 had follow-up PTCR and 141 had 'any event'. RA is weakly associated with an increased risk of all-cause mortality, adjusted risk ratio = 1.3 (95% CI: 0.7–2.3). There is a stronger association between RA and an increased risk of CV death, adjusted HR = 1.9 (95% CI: 0.8–4.7); however, this is not significant. There is no apparent association between case/control status and the percentage of individuals who had non-fatal CV events during the follow-up period.

As expected, other factors were associated with increased mortality. Based on bivariate Cox proportional hazards models including all subjects, the presence of involved vessels is associated with an increase in risk of death (risk ratio = 2.9, 3.0 and 4.6 for 1, 2 and 3 vessels, respectively; *P *= 0.06). Also, history of diabetes (risk ratio = 1.9; *P *= 0.05) and age is associated with an increase in risk of death (risk ratio per 1 year age increase = 1.07; *P *< 0.001).

### Survival plots

**Figure 1:**
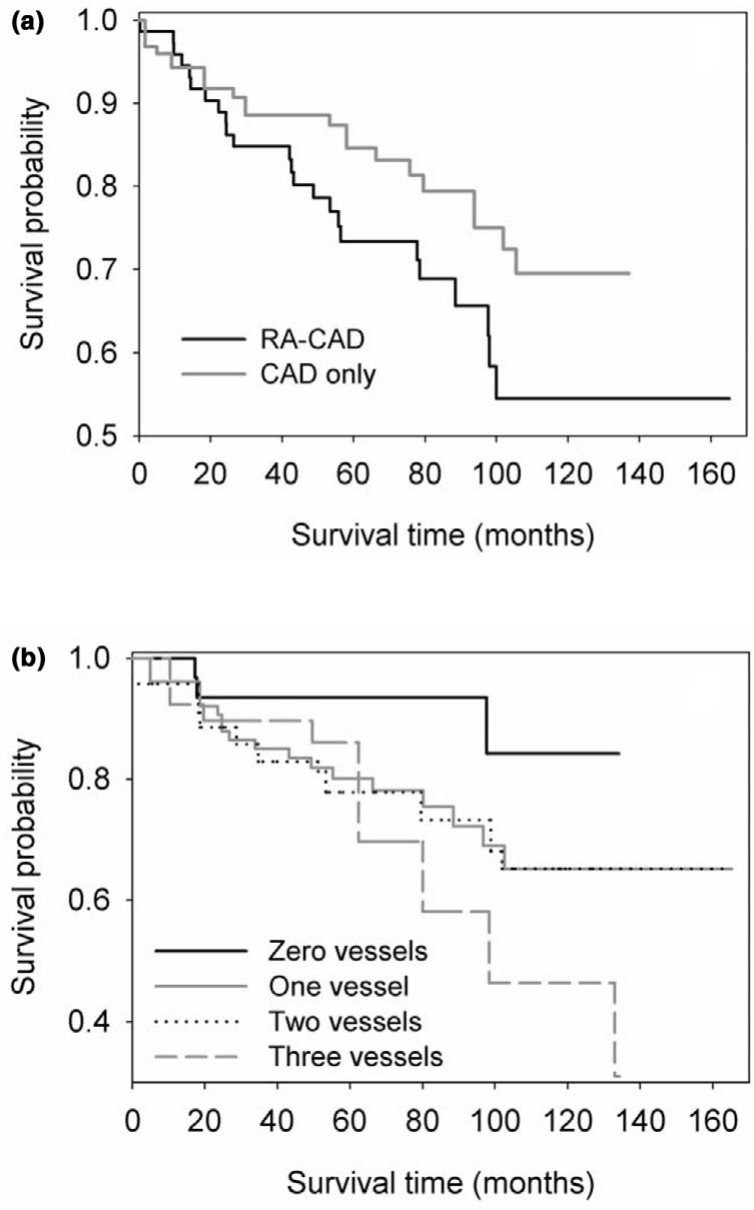
Kaplan-Meier survival curves in CAD patients. Curves include all subjects with CAD classified according to pre-existent RA and to the number of diseased coronary vessels. **(a) **Survival probability was lower in patients with RA (*P *= 0.097) and **(b) **in patients with three affected vessels (*P *= 0.059).

Figure 1a shows Kaplan-Meier plots of survival by case/control status. Survival probability for patients with RA and CAD was lower than that for patients with CAD only (*P *= 0.10). Figure 1b shows Kaplan-Meier plots of survival by number of diseased vessels. Survival probability was lowest for patients with three diseased vessels and was progressively higher as the number of diseased vessels decreased (*P *= 0.06).

### CD4^+^CD28^null ^T cells

**Figure 2:**
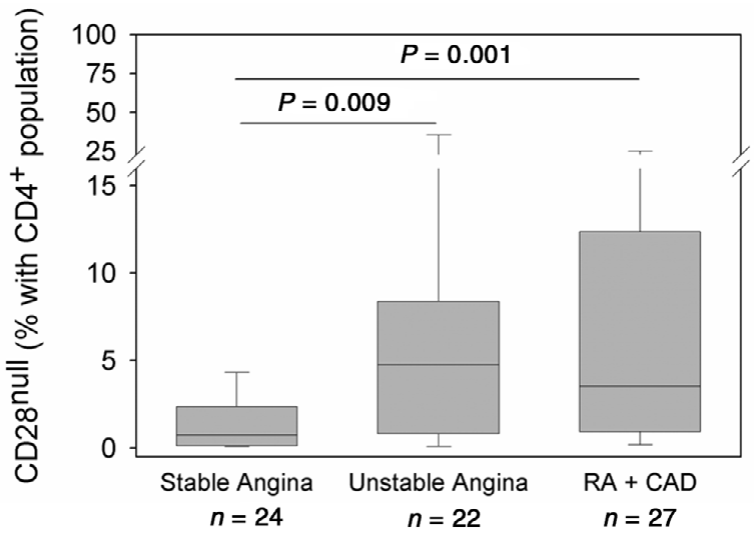
Expansion of non-classic CD4^+^CD28^null ^T cells in patients with RA and CAD. Frequencies of CD4^+^CD28^null ^T cells were determined by flow cytometry. Data are presented as box plots with medians, 25th and 75th percentiles as boxes and 10th and 90th percentile as whiskers. CD4^+^CD28^null ^T cells are infrequent in donors with stable angina and are significantly expanded in patients with unstable angina (*P *= 0.009). Patients with RA and CAD resemble patients with plaque instability and differ from those with stable angina (*P *= 0.001).

As previously reported, frequencies of CD4^+^CD28^null ^T cells were low in individuals with stable angina (median: 0.7%) [6]. In contrast, individuals with unstable coronary syndromes (without RA) had an almost sevenfold expansion of CD4^+^CD28^null ^T cells (median: 4.8%, *P *= 0.009 for stable vs unstable angina comparison). A similar expansion of CD4^+^CD28^null ^T cells was found in patients with RA and CAD (median: 3.5%; 25th percentile: 0.9%; 75th percentile: 12.4%). Frequencies of proinflammatory CD4^+^CD28^null ^T cells were not significantly different between patients with unstable angina (without RA) and those with RA and CAD. Although the patients with RA did not have symptoms of acute plaque instability at the time of the immunological test, the majority of them (81.5%) had a history of unstable angina. The difference between the patients with RA and CAD and stable angina controls was highly significant (*P *= 0.001) (Figure 2).

## Discussion

Our results show that patients with RA have more advanced coronary atherosclerosis at the time of CAD diagnosis compared with patients without RA (Table 2). This occurs independently of the traditional CV disease risk factors. More importantly, this results in a trend towards increased frequency of CV death for patients with RA.

In the general population, the presence of advanced atherosclerosis on angiography is predictive of a worse prognosis [32]. The extent of atherosclerosis determined by angiography has not been studied in RA. Indirect evidence of accelerated atherosclerosis in RA comes from studies using carotid artery intima medial thickness as a marker of atherosclerotic burden and vascular risk [19,33,34]. Increased intima-media thickness was independent of traditional CV risk factors but was related to RA disease activity [20], duration and severity [19]. Data presented here suggest that the acceleration of atherosclerotic disease in RA holds for multiple vascular beds, lending support to a systemic disease mechanism.

### Excess CV morbidity in RA

Patients with RA have a significantly higher prevalence of angina pectoris [34]. Also, women with RA have a significantly increased risk of myocardial infarction compared with those without RA [18]. This excess of CV disease in RA cannot be explained by the traditional Framingham risk factors [35] and probably arises from the underlying disease and/or its treatment. There is no evidence that disease-modifying antirheumatic drug (DMARD) therapy increases mortality in RA [36]. Corticosteroids can cause dyslipidemia, hyperglycemia and hypertension but may also control inflammation in RA. Studies have attempted to define the impact of steroids on mortality in RA but the results are inconsistent [23,25]. DMARD treatment can actually improve the outcome in RA. Choi and colleagues [36] have demonstrated that methotrexate-treated patients had a 70% reduction in CV deaths compared with those who did not receive disease-modifying therapy. Other DMARDs such as sulfasalazine, penicillamine, hydroxychloroquine, and gold did not confer this protection. Thus, the RA disease process itself likely contributes to accelerated CAD.

The inflammatory mechanisms in RA may enhance atherogenesis in several ways. C-reactive protein, a useful marker of disease activity, is elevated in RA and has prognostic value [37]. It may also participate directly in endothelial injury by sensitizing endothelial cells to T-cell mediated cytotoxicity [10]. Circulating cytokines in RA, such as TNF-α, result in endothelial activation and up-regulation of adhesion molecules [38]. Indeed, endothelial dysfunction is frequently present in RA patients, even in the absence of identifiable CV risk factors [39] and improves with anti-TNF-α therapy [40]. Cytokines will also non-specifically activate monocytes and other cells of the innate immune system. RA is characterized by the expansion of autoreactive T-cell clones that typically lack CD28 [11]. The frequency of such CD4^+^CD28^null ^T cells correlates with disease severity with respect to erosive progression [41] and extra-articular manifestations. The frequency in the RA with CAD cohort (median 3.5%) was higher than in historical controls of patients with RA and absence of extra-articular manifestations [11], suggesting that CV comorbidity in RA is correlated with disease severity and that CD4^+^CD28^null ^T cells may be involved in the CV complications of RA. CD4^+^CD28^null ^T cells have been directly implicated in the pathogenesis of coronary artery disease [6]. Persistent activation of such autoreactive cells in RA may result in a vicious cycle of cytokine release, mononuclear cell activation and tissue injury. However, we cannot exclude the possibility that the high CD4^+^CD28^null ^T cells levels in RA with CAD patients is reflective of an increased RA disease severity in these patients. Addressing this issue further will require comparing RA patients that are matched for disease severity but are disparate for CAD.

### Study limitations

We adjusted for traditional Framingham risk factors that are commonly considered in epidemiological studies of CV disease. However, factors such as cigarette smoking could possibly have a synergistic effect with chronic inflammation due to RA, resulting in increased atherogenesis. In our study, detailed quantitative data on amounts of tobacco used were not available. Also, data regarding other risk factors, such as family history and body mass index, were not available. Elevated levels of homocysteine could represent another risk factor for atherosclerosis in RA. Methotrexate, a commonly used disease-modifying agent, inhibits dihydrofolate reductase and reduces levels of folate, which in turn increases levels of homocysteine [42,43]. The impact of folic acid supplementation on homocysteine levels and CV disease in patients with RA is unknown. Data on homocysteine levels were not available in our study. Finally, it is possible that our findings are a reflection of patients with RA being less symptomatic from CAD than the general population, therefore resulting in later presentation when the disease is more advanced.

Despite more severe CAD at the time of clinical presentation, the prevalence of non-fatal CV events was not increased in patients with RA. It is possible that significant differences between cases and controls were not detected because of the size of the cohorts studied and also that CV events may have been underestimated due to the retrospective nature of the study.

## Conclusion

In summary, our results demonstrate that patients with RA have a greater burden of coronary atherosclerosis at their first angiogram that is independent of traditional CV risk factors. This may be due, at least in part, to the expansion of nonclassic CD4^+ ^T cells that have previously been implicated in the pathogenesis of CAD [6,9].

## Abbreviations

ACS = acute coronary syndrome; CABG = coronary artery bypass graft; CAD = coronary artery disease; CV = cardiovascular; HR, hazard ratio; DMARD = disease-modifying antirheumatic drugs; MI = myocardial infarction; OR = odds ratio; PTCR = percutaneous transluminal coronary revascularization; RA = rheumatoid arthritis.

## Competing interests

The authors declare that they have no competing interests.

## Authors' contributions

KJW carried out chart reviews, performed the immunological assays, participated in study design and drafted the manuscript. PDK carried out chart reviews and data collection. RLF participated in study design and manuscript preparation. JFL performed the statistical analyses. SLK carried out data interpretation and participated in study design. JJG participated in study design, interpretation of data and manuscript preparation. CMW conceived the study, participated in its design and coordination and helped to draft the manuscript. All authors read and approved the final manuscript.

## Figures and Tables

**Table 1:** Patient demographics

Variable	RA + CAD (*n *= 75)	CAD (*n *= 128)	*P*^*a*^
Age, median (Q1, Q3)	66.4 (60.7, 71.7)	66.7 (59.8, 71.4)	0.66
Sex, *n *(%)			0.63
Male	39 (52)	71 (55)	-
Female	36 (48)	57 (45)	-
History of diabetes, *n *(%)	14 (19)	24 (19)	0.99
History of hypertension, *n *(%)	28 (37)	51 (40)	0.72
History of hyperlipidemia, *n *(%)	11 (15)	31 (24)	0.10
History of smoking, *n *(%)	11 (15)	23 (18)	0.54
Age at RA onset, year	55.3 ± 12.7	-	
RA disease duration, year	17.6 ± 11.0	-	
Rheumatoid factor positive, *n *(%)	68 (90.7)	-	
Nodular disease, *n *(%)	40 (53.3)	-	
Extra-articular disease, *n *(%)	16 (21.3)	-	
Steroid use, *n *(%)	55 (73.3)	-	
DMARD use, *n *(%)	68 (90.7)	-	

^a^*P *values are from chi-square tests, except for age, where it is from a Wilcoxon rank-sum test. CAD, coronary artery disease; DMARD, disease-modifying antirheumatic drug; RA, rheumatoid arthritis.

**Table 2:** Angiography results

	RA + CAD (*n *= 75)	CAD (*n *= 128)
Number of diseased vessels, *n *(%)		
0	3 (4)	30 (23)
1	36 (48)	43 (34)
2	18 (24)	33 (26)
3	18 (24)	22 (17)

CAD, coronary artery disease; RA, rheumatoid arthritis

**Table 3:** Ordinal logistic regression models for the number of diseased vessels

Variable	Unadjusted OR (95% CI)	*P*	Adjusted OR^a^(95% CI)	*P*
RA diagnosis	1.73 (1.03, 2.91)	0.04	1.97 (1.15, 3.36)	0.01
History of hyperlipidemia	NA	NA	2.56 (1.35, 4.85)	0.004
Age (per year increase)	NA	NA	1.05 (1.02, 1.07)	0.002
Sex (female relative to male)	NA	NA	0.40 (0.23, 0.67)	0.001

^a^Adjusted model is the result of a forward selection procedure, and adjustment was made for age, sex and history of hyperlipidemia.

CI, confidence interval; NA, not applicable; OR, odds ratio; RA, rheumatoid arthritis.

**Table 4:** Summary of events during follow-up^a^

Event	RA + CAD (*n *= 75), *n *(%)	CAD (*n *= 128), *n *(%)	Unadjusted HR (95% CI)	*P*	Adjusted HR^b ^(95% CI)	*P*
CV death	13 (17)	9 (7)	2.22 (0.95, 5.20)	0.06	1.94 (0.80, 4.69)	0.14
Death	24 (32)	23 (18)	1.63 (0.92, 2.89)	0.10	1.29 (0.72, 2.32)	0.39
CABG during follow-up	18 (24)	34 (27)	0.87 (0.49, 1.53)	0.62	0.80 (0.44, 1.44)	0.45
MI during follow-up	28 (37)	43 (34)	1.08 (0.67, 1.73)	0.77	0.79 (0.48, 1.28)	0.34
PTCR during follow-up	27 (36)	47 (37)	0.78 (0.43, 1.41)	0.41	0.69 (0.37, 1.27)	0.23
Any event	53 (71)	88 (69)	1.19 (0.84, 1.68)	0.34	1.05 (0.73, 1.50)	0.81

^a^Raw counts and Cox model results for various follow-up endpoints.

^b^Adjusted models include number of vessels and hyperlipidemia in the model as covariates.

CABG, coronary artery bypass surgery; CAD, coronary artery disease; CI, confidence interval; CV, cardiovascular; HR, hazard ratio; MI, myocardial infarction; PTCR, percutaneous transluminal coronary.
